# Chemotherapeutic efficacies of a clofazimine and diminazene aceturate combination against piroplasm parasites and their AT-rich DNA-binding activity on *Babesia bovis*

**DOI:** 10.1038/s41598-017-14304-0

**Published:** 2017-10-24

**Authors:** Bumduuren Tuvshintulga, Mahmoud AbouLaila, Thillaiampalam Sivakumar, Dickson Stuart Tayebwa, Sambuu Gantuya, Khandsuren Naranbaatar, Aki Ishiyama, Masato Iwatsuki, Kazuhiko Otoguro, Satoshi Ōmura, Mohamad Alaa Terkawi, Azirwan Guswanto, Mohamed Abdo Rizk, Naoaki Yokoyama, Ikuo Igarashi

**Affiliations:** 1 National Research Center for Protozoan Diseases, Obihiro University of Agriculture and Veterinary Medicine, Inada-cho, Obihiro, Hokkaido 080-8555 Japan; 2 Laboratory of Molecular Genetics, Institute of Veterinary Medicine, Ulaanbaatar, Mongolia; 3 Department of Parasitology, Faculty of Veterinary Medicine, University of Sadat City, Sadat City, 32511 Minoufiya Egypt; 4 Laboratory of Arachno-Entomology and Protozoology, Institute of Veterinary Medicine, Ulaanbaatar, Mongolia; 5 Research Center for Tropical Diseases, Kitasato Institute for Life Sciences, Kitasato University, Tokyo, Japan; 6 Frontier Research Center for Advanced Material and Life Science, Department of Orthopedic Surgery, School of Medicine, Hokkaido University, Kita 21, Nishi 11, Kita-ku, Sapporo, Hokkaido, 001-0021 Japan; 7 Department of Internal Medicine and Infectious Diseases, Faculty of Veterinary Medicine, Mansoura University, Mansura, 35516 Dakahlia Egypt

## Abstract

Recently, we reported that clofazimine (CF) has an anti-piroplasm activity, but it could not completely eliminate parasites in the host. The currently available anti-piroplasm drug, diminazene aceturate (DA), has sometimes been reported to have toxic side effects. In the present study, we evaluated the combination treatment with CF and DA against piroplasms both *in vitro* and *in vivo*. Additionally, mRNA level and DNA amounts were analyzed in CF‒ and DA‒treated *Babesia bovis* by a qPCR. The CF–DA combination had additive effects on *Babesia bovis*, *B*. *bigemina*, and *B*. *caballi* and synergistic effects on *Theileria equi*. The CF–DA combination chemotherapies against *B. microti* in mice were more potent than their monotherapies. In the CF‒ and DA‒treated *B. bovis*, CF dose-dependently down-regulated mRNA level and DNA amounts of extranuclear genes (AT-rich featured), whereas DA down-regulated only DNA amounts of extranuclear genes, but those of nuclear genes were slightly down- or up-regulated by CF and DA. In conclusion, the CF–DA combination has a higher efficiency against piroplasms than CF or DA monotherapies. CF and DA might have an AT-rich DNA-binding activity. All results suggest that the CF–DA combination chemotherapy will be a better choice to treat piroplasmosis instead of DA monotherapy.

## Introduction

Bovine babesiosis and equine piroplasmosis cause a huge economic loss in the animal industry worldwide, especially in the tropical and subtropical regions^1,2^. The currently available anti-piroplasm drugs, diminazene aceturate (DA) and imidocarb dipropionate, have sometimes been reported to have toxic side effects^3^. In addition, DA-resistant *Babesia gibsoni* and the development of imidocarb dipropionate–resistant *Theileria equi* have been reported^4,5^. Therefore, less-toxic, low-cost and potent anti-piroplasm chemotherapies are urgently needed.

We recently reported that clofazimine (CF) has a potent inhibitory effect on the growth of piroplasms, but CF could not completely eliminate *B*. *microti* in mice^6^. CF is an antibiotic used in combination chemotherapies against leprosy^7^. It has been reported that CF hardly leads to the development of resistance^8^, but Zhang *et al*.^9^ recently reported that several mutations in genes are associated with CF resistance in bacteria.

Combination chemotherapy has been recommended against drug-resistant tumor cells and protozoan and bacterial pathogens^10–13^. Similarly, combination chemotherapy might be useful to reduce the dosages of individual drugs, thereby reducing their toxic side effects^10^. In combination therapy, drugs sharing the same mode of action tend to yield a synergistic effect, which can be defined as the greater effect of two drugs in combination than the sum of each drug when acting separately, or an additive effect, in which the effect of two drugs in combination is equal to the sum of each drug when acting separately^10,11^. Previous studies reported that both CF and DA have an AT-rich DNA-binding activity on *Mycobacterium* species and *Trypanosoma cruzi*, respectively^14,15^. AT-rich DNA-binding drugs influence the RNA synthesis and DNA replication by selectively interacting with AT-rich regions in DNA. Several AT-rich DNA-binding drugs are known to possess anti-bacterial, anti-protozoal and anti-cancer activities^14–16^. In apicomplexan parasites, AT-rich regions are commonly found, especially in the mitochondrial and apicoplast genomes, making them an attractive target for AT-rich DNA-binding drugs^15,17^. Moreover, toxic side effects of CF and DA were found to be non-overlapping^3,7^. The objective of our study is therefore to evaluate the CF–DA combination as an effective treatment strategy that can reduce the dose of DA for avoiding strong toxic side effects and without affecting the clinical outcome.

In the present study, we investigated the effect of CF–DA combination on the *in vitro* growth of *B. bovis*, *B. bigemina*, *B. caballi* and *T. equi*, and its chemotherapeutic activities against *B. microti* in mice. In addition to growth inhibition assays, AT-rich DNA-binding activities were studied to find out the mode of CF and DA actions in *B. bovis* using a quantitative PCR assay (qPCR).

## Results and Discussion

In the present study, we considered the sum of the fractional inhibitory concentration ∑FIC index as a criterion to determine the effects of combination drugs, as it is the most commonly used method^18–20^. Additive effects of CF–DA combinations were observed on *B. bovis*, *B. bigemina*, and *B. caballi*, except for a combination (1:4) that showed synergism on *B*. *caballi* (Tables 1‒3). All combinations had synergistic effect on *T. equi* (Table 4). The 50% inhibitory concentration (IC_50_) values of CF in combinations showed a higher inhibitory effect compared to the IC_50_ value of CF (mono), while the IC_50_ values of DA in combinations sometimes showed a higher or at least a similar inhibitory effect as compared to its IC_50_ of DA (mono) (Tables 1‒4). The lowest IC_50_ values of CF (mono) and DA (mono) were observed at 2.88 ± 0.18 and 0.09 ± 0.003 µM on *T. equi* and *B. caballi*, respectively, and this might be the reason for the observed synergistic effect. Certainly, the additive and synergistic effects in combinations were indicated by significantly lower IC_50_ values of drugs in combinations than their IC_50_ value of mono-drugs. Since the additive and synergistic effects of combination drugs *in vitro* warranted *in vivo* experiments^21,22^, the CF–DA combination was evaluated in mice to determine whether this combination would be useful in reducing the dose of DA without altering the therapeutic efficacy in *B. microti* infection.

**Table 1:** Inhibitory effects of CF/DA and their combinations on *B*. *bovis*.

Mono and combination	CF relative mean of IC_50_ ± SD (µM)	DA relative mean of IC_50_ ± SD (µM)	ΣFICs, Interaction	
5:0	*8.24 ± 0.95	—		
4:1	6.82 ± 0.77	0.34 ± 0.04	1.18	**Additive effect**
3:2	5 ± 0.9	0.67 ± 0.12	1.3	**Additive effect**
2:3	3.38 ± 1.03	0.99 ± 0.28	1.44	**Additive effect**
1:4	1.24 ± 0.11	1 ± 0.09	1.19	**Additive effect**
0:5	—	*0.96 ± 0.29		

*IC_50_ value of mono-drug.

**Table 2:** Inhibitory effects of CF/DA and their combinations on *B*. *bigemina*.

Mono and combination	CF relative mean of IC_50_ ± SD (µM)	DA relative mean of IC_50_ ± SD (µM)	ΣFICs, Interaction	
5:0	*5.73 ± 0.57	—		
4:1	5.77 ± 0.33	0.29 ± 0.01	1.08	**Additive effect**
3:2	4.75 ± 0.12	0.63 ± 0.01	1	**Additive effect**
2:3	5.12 ± 0.59	1.53 ± 0.18	1.31	**Additive effect**
1:4	3.16 ± 0.43	2.28 ± 0.01	1.18	**Additive effect**
0:5	—	*3.64 ± 0.44		

*IC_50_ value of mono-drug.

**Table 3:** Inhibitory effects of CF/DA and their combinations on *B*. *caballi*.

Mono and combination	CF relative mean of IC_50_ ± SD (µM)	DA relative mean of IC_50_ ± SD (µM)	ΣFICs, Interaction	
5:0	*7.95 ± 0.08	—		
4:1	2.28 ± 0.35	0.12 ± 0.02	1.62	**Additive effect**
3:2	1.21 ± 0.16	0.16 ± 0.02	1.93	**Additive effect**
2:3	0.36 ± 0.06	0.11 ± 0.01	1.27	**Additive effect**
1:4	0.09 ± 0.01	0.07 ± 0.001	0.79	**Synergism**
0:5	—	*0.09 ± 0.003	—	

*IC_50_ value of mono-drug.

**Table 4:** Inhibitory effects of CF/DA and their combinations on *T*. *equi*.

Mono and combination	CF relative mean of IC_50_ ± SD (µM)	DA relative mean of IC_50_ ± SD (µM)	ΣFICs, Interaction	
5:0	*2.88 ± 0.18	—		
4:1	2.2 ± 0.23	0.11 ± 0.01	0.88	**Synergism**
3:2	1.77 ± 0.08	0.23 ± 0.01	0.86	**Synergism**
2:3	1.17 ± 0.04	0.35 ± 0.01	0.79	**Synergism**
1:4	0.58 ± 0.02	0.47 ± 0.01	0.71	**Synergism**
0:5	—	*0.92 ± 0.74	—	

*IC_50_ value of mono-drug.

**Figure 1:**
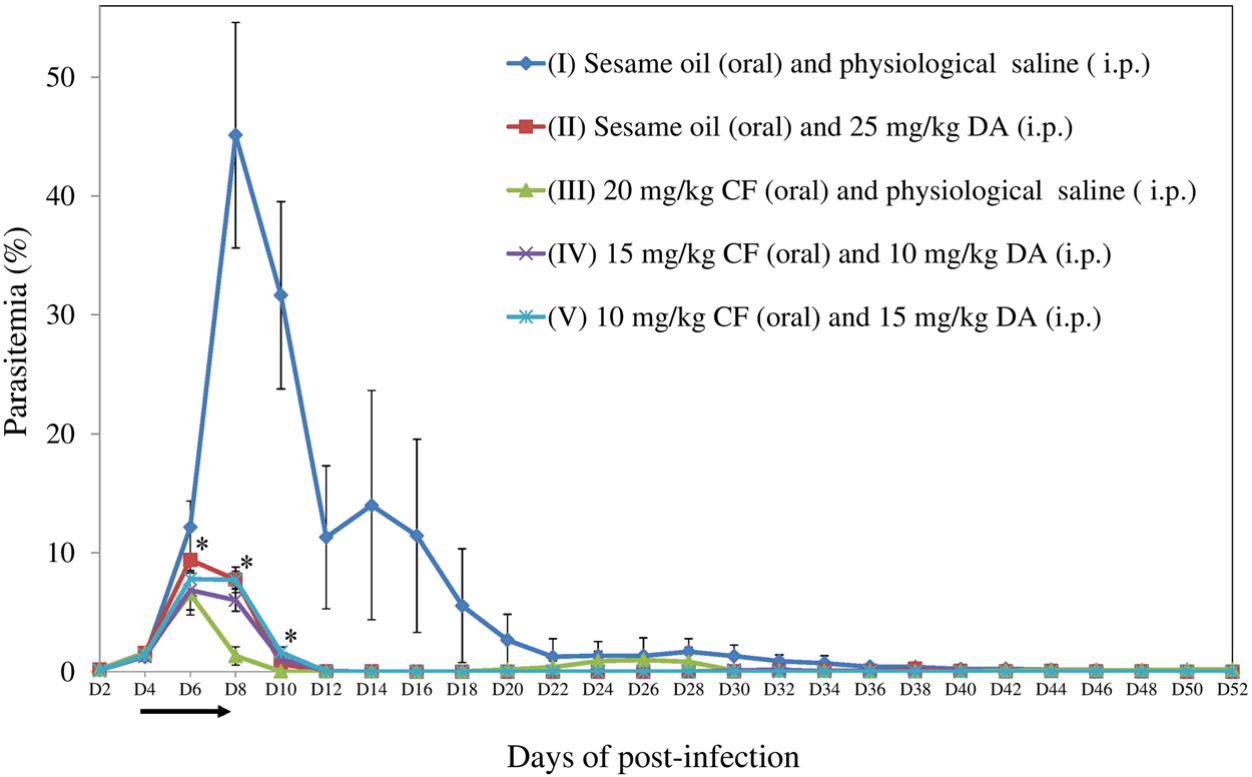
Growth of *B. microti* in untreated (I), 25 mg/kg DA (II)–, 20 mg/kg CF (III)–, 15 mg/kg CF and 10 mg/kg DA combination (IV)–, and 10 mg/kg CF and 15 mg/kg DA combination (V)-treated mice for 52 days. The arrow indicates 5 consecutive days of treatment. Asterisks indicate statistically significant (*P* < 0.05) differences of parasitemia based on unpaired *t-*test analysis.

In agreement with the previous study^6^, the effect of 20 mg/kg CF was comparable to and sometimes higher than that of 25 mg/kg DA. Therefore 25 mg/kg, the highest dosage, was considered as a baseline for selection of dosages in combination therapies in mice experiment. In addition, unequal ratio dosages in combinations in the *in vivo* inhibition assay is similar to that in the *in vitro* inhibition assay. The growth of *B. microti* was significantly inhibited in all treated groups (II-25 mg/kg DA, III-20 mg/kg CF, IV-15 mg/kg CF and 10 mg/kg DA, and V-10 mg/kg CF and 15 mg/kg DA) as compared to the untreated group (I-0.2 ml sesame oil and physiological saline) (Fig. 1). A statistically significant lower difference (*P* < 0.05) of parasitemia was observed in groups II or V and III or IV on days 8–10 and 6–10 post-infection, respectively, as compared to its control (I). On day 10 post-infection, there was no statistically significant difference between groups II and V, whereas group IV showed a statistically significant lower difference of parasitemia as compared to group II, but group III showed a statistically significant lower difference of parasitemia as compared to groups IV and V on days 8–10 post-infection. However, a relapse of *B. microti* infection was observed on days 22‒30 post-infection in group III. The peak of parasitemia was observed on day 8 post-infection in group I, while the parasitemia in groups II, III, IV, and V reached its peak on day 6 post-infection. As compared to group I, the peak of parasitemia in groups II, III, IV, and V was inhibited at 79.2, 85.5, 84.8, and 82.7%, respectively. Notably, the parasite was not detected within 10,000 RBCs in Giemsa-stained blood smears from CF–DA combination treatment groups from days 44 (group IV) and 34 (group V) to 52 post-infection, while the parasite was detected in groups I and III until day 50 post-infection and in group II until day 48 post-infection. On day 52, no parasite was detected in all mice by microscopy.

**Figure 2:**
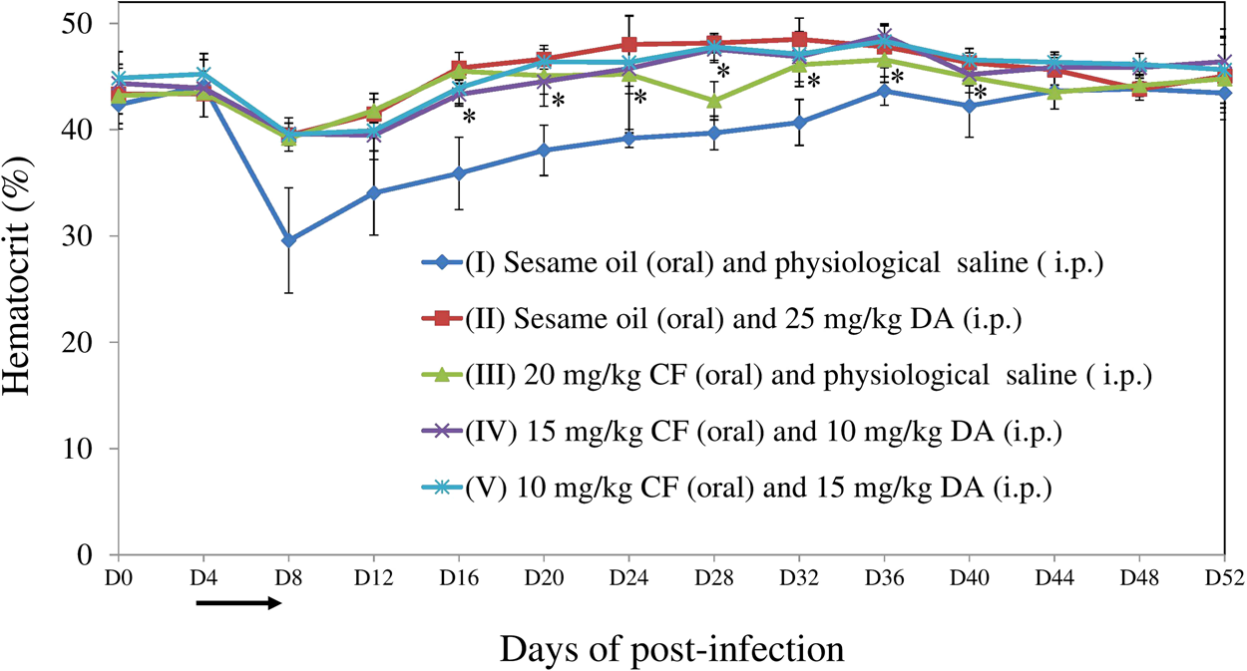
Hematocrit values in untreated, 25 mg/kg DA–, 20 mg/kg CF–, 15 mg/kg CF and 10 mg/kg DA combination–, and 10 mg/kg CF and 15 mg/kg DA combination-treated mice. The arrow indicates 5 consecutive days of treatment. Asterisks indicate statistically significant (*P* < 0.05) differences of hematocrit values based on unpaired *t-*test analysis.

As for the monitoring of blood parameters in mice, higher statistically significant differences (*P* < 0.05) of hematocrit (as an anemia index) values were observed in group II on days 16–32, group III on days 16, 20, and 32, group IV on days 20–40, and group V on days 16–40 post-infection than in group I (Fig. 2).

Taken together, CF–DA combination chemotherapies (84.8 and 82.7%) showed a slightly higher and lower inhibition of the peak of parasitemia than DA (79.2%) and CF (85.5%) monotherapies, respectively. Especially, a combination chemotherapy (IV) showed a significantly lower parasitemia on day 10 post-infection as compared to the DA monotherapy. Furthermore, the early clearance of *B. microti* was observed in blood smears from combination treatment groups (from days 34‒ or 44‒52 post-infection) as compared with groups treated with DA (on days 50 and 52 post-infection) or CF (only on day 52 post-infection) monotherapy when disregarding the low dosage (20 mg/kg) of the CF monotherapy. In addition, the hematocrit indexes recovered quickly in the combination treatment groups (on days 16‒ or 20‒40) as compared to that in the CF monotherapy group (on days 16, 20, and 32 post-infection). Therefore, CF–DA combination therapy might perform better in terms of early clearance and clinical outcome as compared to CF and DA monotherapies.

**Figure 3:**
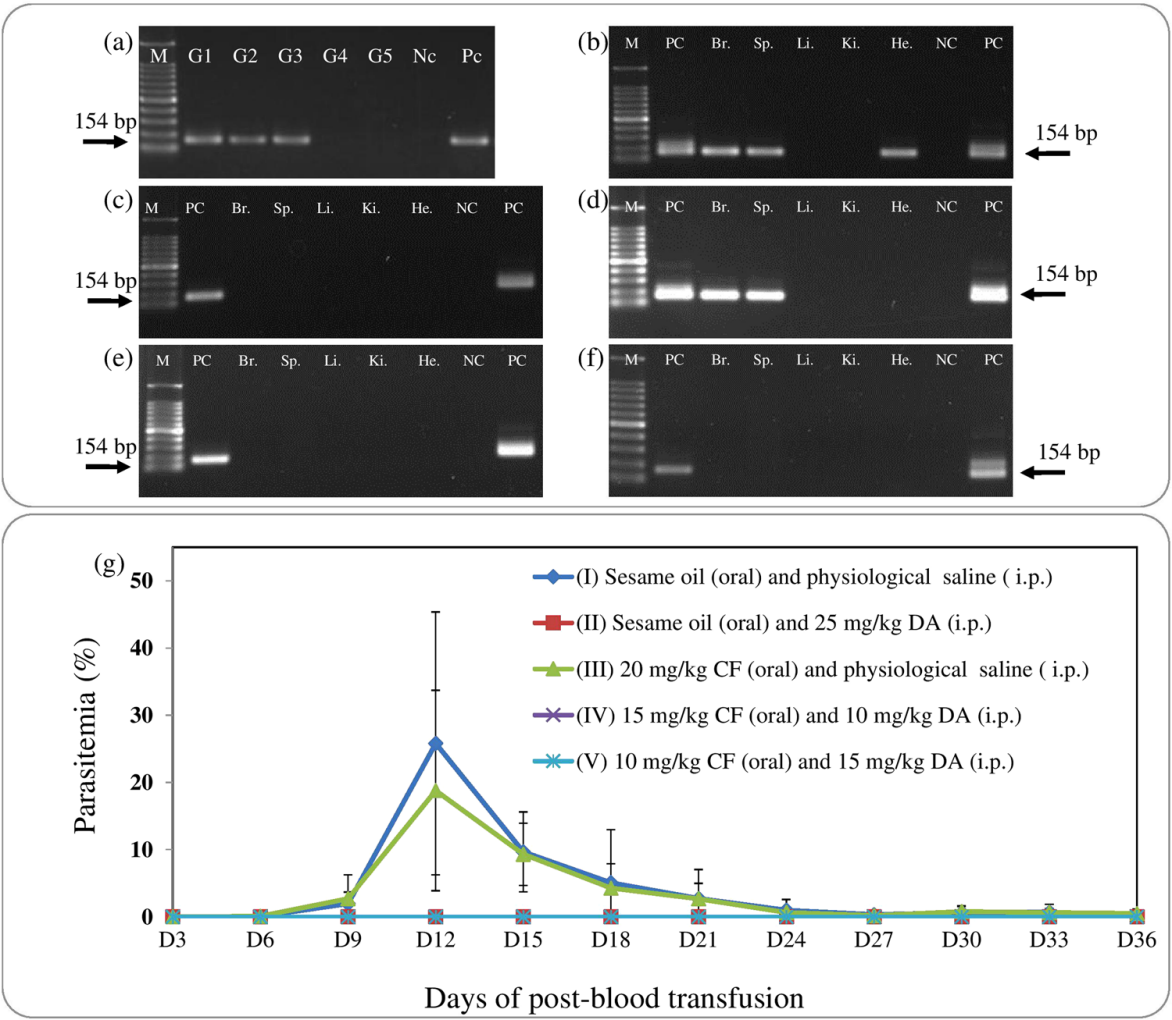
PCR detection of *B. microti* from CF‒ or DA‒treated mice on day 52 post-infection. (**a**) in blood DNA samples: G1, untreated; G2, 25 mg/kg DA; G3, 20 mg/kg CF; G4, 15 mg/kg CF and 10 mg/kg DA combination; G5, 10 mg/kg CF and 15 mg/kg DA combination. (**b**–**f**) in tissue  DNA samples: M, 100 bp size marker; PC, positive control; Br., brain; Sp., spleen; Li., liver; Ki., kidney; He., heart, NC, negative control. The double bands observed with some of the positive controls represent amplicons of the first and second PCR. Only a single band was observed when the concentration of template DNA was reduced (data not shown). (**g**) Infectivity of *B. microti* in RBC-transfused mice. Parasitemia was calculated by counting infected RBCs among 500 RBCs.

After *in vivo* inhibition assay, *B. microti* was detected in blood DNA samples from groups I, II, and III by PCR assay, while it was not detected in groups IV and V (Fig. 3a). In the case of group I or III, *B. microti* was detected in the brain, spleen, and heart or in the brain and spleen tissue DNA samples, respectively (Fig. 3b and d). Whereas, parasites were not detected in tissue samples from groups II, IV, and V (Fig. 3c,e and f). As for infectivity of parasites on day 52 post-infection, the growth of *B. microti* was observed in RBC-transfused mice from groups I and III, while the parasite did not grow in RBC-transfused mice from groups II, IV, and V (Fig. 3g). These results showed that combination chemotherapy could completely eliminate parasite in the host, and this deduction was supported by early clearance of *B. microti* in blood smears from combination‒treated mice.

Overall, CF–DA combination chemotherapies are more potent than their monotherapies, despite different dosage regimens used in the both *in vitro* and *in vivo* inhibition assays, therefore, combination chemotherapy likely reduces toxic side effects on the host. However, further studies should be conducted to investigate possible toxic side effects associated with CF–DA combination therapy to confirm our hypothesis.

For the combination of two or more drugs, molecular targets should be determined^10,11,19,20^. However, prediction of the drugs targets is quite difficult to determine quantitatively in terms of synergism and additive effects^10^. One of the mode of CF and DA actions is a DNA-binding activity that inhibits bacterial and protozoal proliferation, respectively^14,15^. On the other hand, genomic investigations found that *B. bovis* mitochondrial and apicoplast genomes have AT-rich featured regions in their DNA that is similar to the minor groove of DNA in bacteria^17^. With this understanding, we investigated the DNA-binding activity of CF and DA on *B. bovis* using qPCR to analyze the mRNA level and DNA amounts of specific genes in nuclear, mitochondrial, and apicoplast genomes. Before qPCR assay, we confirmed that CF and DA do not damage the parasite plasma membrane-cytoskeleton systems based on the observations of timely morphological changes of parasites. We selected 1, 1.5, and 2 µM of CF and 0.04, 0.3, and 0.75 µM of DA as testing concentrations based on 25, 50, and 75% inhibitory concentration (IC_25_, IC_50_, and IC_75_) values (Table 5) determined using the 2-day inhibition assay. A typical shape including a pear, ring, and degenerative form (Fig. S1a) of parasite was observed in all cultures at 0.5, 1, 2, 4, 6, 8, 12, 24, 36, and 48 hours, while the number of parasites was decreased by DA or CF treatment (data not shown). In the case of CF-treated *B. bovis*, no statistically significant difference was observed between single- and pair-cell stages as compared to the dimethyl sulfoxide (DMSO)-treated parasites (Fig. S1b and c). On the other hand, statistically significant differences of high number of single-cell stage including ring and degenerative forms were observed in 0.75 µM DA-treated cultures at 36 hours (66%, *P* < 0.05) and 48 hours (76%, *P* < 0.01) as compared to that in Milli-Q water (M-QW)-treated cultures (51% and 45%, respectively) (Fig. S1b and c). However, no specific morphological changes were observed in DA- and CF-treated cultures as compared to that in their untreated control cultures (Figs S2‒5).

**Table 5:** The IC_25_, IC_50_, and IC_75_ values of CF and DA on *B*. *bovis* using a 1- or 2-day inhibition assay.

Drugs		Changing media		Assays	Inhibitory concentrations (µM)		
		Day 0	Day 1		IC_25_ ± SD	IC_50_ ± SD	IC_75_ ± SD
CF	1-day	w/o drug	w drug	Microscopic examination	ND*	3.42 ± 0.72	10.53 ± 4.08
	2-day	w drug	w drug	Microscopic examination	1.07 ± 0.07	1.48 ± 0.07	1.94 ± 0.25
				Fluorescence-based assay	1.86 ± 0.21	2.61 ± 0.19	4.00 ± 0.0
DA	1-day	w/o drug	w drug	Microscopic examination	ND*	6.46 ± 1.9	19.23 ± 1.33
	2-day	w drug	w drug	Microscopic examination	0.04 ± 0.02	0.27 ± 0.17	0.75 ± 0.02
				Fluorescence-based assay	0.04 ± 0.01	0.50 ± 0.0	1.29 ± 0.31

ND*—not determined; w/o—without; w—with.

Using the 1-day inhibition assay, the IC_50_ and IC_75_ values at 3.42 ± 0.72 and 10.53 ± 4.08 µM of CF or 6.46 ± 1.9 and 19.23 ± 1.33 µM of DA were observed on *B. bovis* (Table 5), and then 4 and 15 µM CF or 6.5 and 19.5 µM DA were selected for mRNA and DNA quantification analysis.

**Figure 4:**
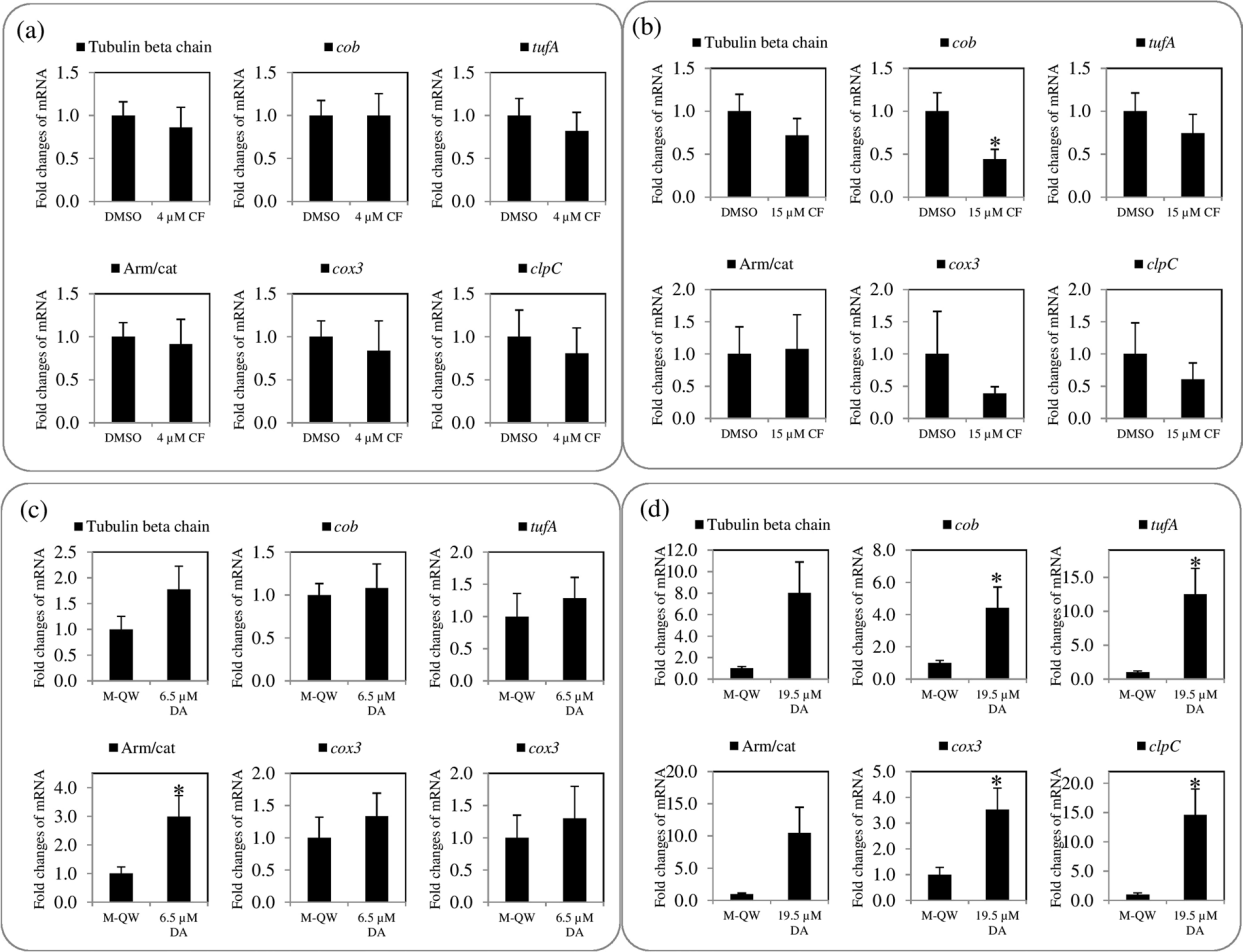
mRNA level in CF- or DA-treated *B. bovis*. (**a**) 4 µM CF-treated and DMSO-treated parasites. (**b**) 15 µM CF-treated and DMSO-treated parasites. (**c**) 6.5 µM DA-treated and Milli-Q water (M-QW)-treated parasites. (**d**) 19.5 µM DA-treated and M-QW-treated parasites. Asterisks indicate statistically significant (^*^ *P* < 0.05) differences of fold changes based on unpaired *t-*test analysis.

The mRNA level of all genes (excluding *cob*) in 4 µM CF-treated *B. bovis* was shown to have slight lower fold changes (Fig. 4a), and that in 15 µM CF-treated *B. bovis* showed down-regulation as compared to those of all genes in DMSO-treated *B. bovis*, except that of the armadillo/beta-catenin-like repeat domain containing protein (Arm/cat) gene with slight up-regulation (Fig. 4b). A comparison between 4 and 15 µM CF-treated groups, the fold changes of all genes’ mRNA levels were regulated in a dose-dependent manner (Fig. 4a and b). However, a statistically significant (*P* < 0.05) difference was observed only on the *cob* mRNA level in 15 µM CF-treated *B. bovis* as a down-regulation (Fig. 4b). This observation might be explained by the high percentage of guanine base (19%) in *cob* as compared to mitochondrial and apicoplast genes (Table S1). The guanine base plays an important role in DNA-binding activity of CF in bacteria^8^. Taken together, CF might more stably bind with appropriate high percentage of guanine base in AT-rich featured DNA fragment of gene like *cob* among investigated genes in the present study. On the other hand, an up-regulation was observed on the mRNA levels of all genes in 6.5 and 19 µM DA-treated parasites as compared to the M-QW-treated. A statistically significant difference was observed on the Arm/cat gene (Fig. 4c) and extranuclear genes (Fig. 4d) in 6.5 and 19.5 µM DA treatments, respectively, but no statistically significant difference was observed on nuclear genes in 19.5 µM DA-treated parasites as compared to its control. The mRNA level of extranuclear genes was dose-dependently regulated in 6.5 and 19.5 µM DA-treated groups. The up-regulation of mRNA level might be relative to the survival of DA-treated parasites as previously reported by Wu *et al*.^16^ who showed that AT-rich DNA-binding ligand induces up-regulation of mRNA level of survivin protein gene in cancer cells. Furthermore, the up-regulation of mRNA of genes is associated with the development of resistance in *Leishmania donovani* and cancer cells^16,23^. Additionally, statistically significant differences of high number of single-cell stages were observed in a comparison between 0.75 µM DA-treated and untreated parasites at 36 and 48 hours, while the CF-treated parasite did not show such differences (Fig. S1b and c), and the single-cells, including ring and degenerative forms, are known to be non-dividing stages. Hence, future study should be focused the development of DA-resistance in *B. bovis* and CF as well. However, it is generally believed that the development of resistance in protozoan parasites is a very slow process^24^.

Based on the results of mRNA quantification analysis of the present investigation and findings of previous studies^14,15^, we hypothesized that a comparison of the differences of fold changes on the mRNA levels of nuclear, mitochondrial, and apicoplast genes between CF- or DA-treated and untreated parasites revealed the association with a DNA-binding activity of these drugs. In addition to that, CF does not inhibit RNA polymerase^8^ and the RNA binder activities of CF and DA are unclear. Therefore, we investigated the DNA amounts of nuclear, mitochondrial, and apicoplast genes between CF- or DA-treated and untreated parasites using a qPCR assay.

**Figure 5:**
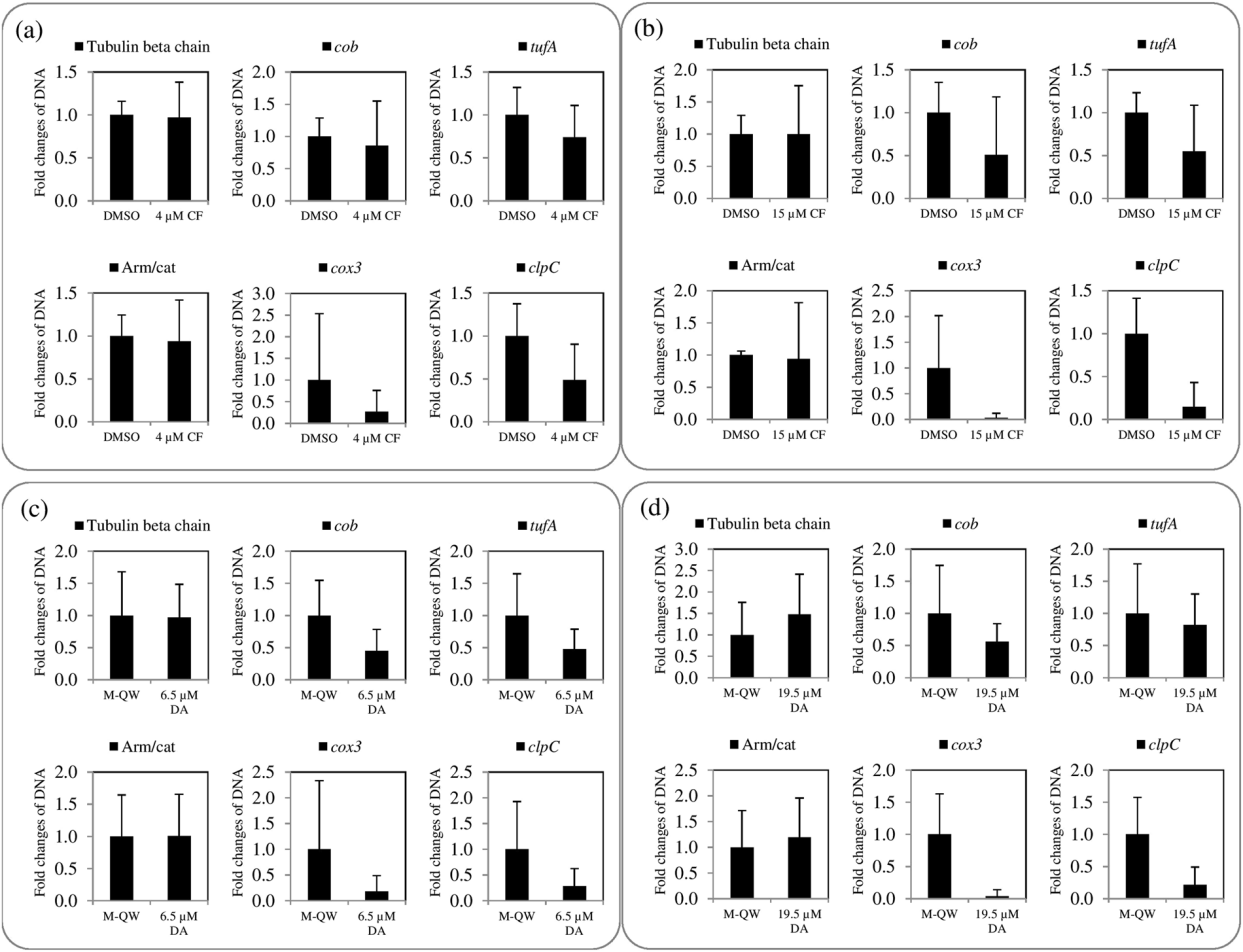
DNA amount in CF- or DA-treated *B. bovis*. (**a**) 4 µM CF-treated and DMSO-treated parasites. (**b**) 15 µM CF-treated and DMSO-treated parasites. (**c**) 6.5 µM DA-treated and Milli-Q water (M-QW)-treated parasites. (**d**) 19.5 µM DA-treated and M-QW-treated parasites.

Regarding DNA quantification analysis, fold changes of DNA amounts of extranuclear genes in the 4 and 15 µM CF-treated parasites were dose-dependently down-regulated as compared to that in DMSO-treated parasites, but that of tubulin beta chain gene and Arm/cat genes were not and slightly regulated, respectively (Fig. 5a and b). Furthermore, the DNA amount of nuclear genes was not regulated, while that of extranuclear genes was down-regulated in 6.5 µM DA-treated parasites as compared to those in M-QW-treated parasites (Fig. 5c). In case of 19.5 µM DA‒treated parasites, an up-regulation was observed on the DNA amounts of nuclear genes, whereas down-regulation was observed on mitochondrial and apicoplast genes as compared to its control (Fig. 5d).

The results of qPCR suggest that CF and DA bind AT-rich featured DNA of mitochondria and apicoplast to inhibit the mRNA and DNA biosyntheses of extranuclear genes in *B. bovis*. In contrast, DA could not inhibit mRNA biosynthesis of all genes, as well as CF and DA might not be toxic to mRNA and DNA biosyntheses of nuclear genes.

In conclusion, the CF–DA combination showed  additive effects on the *in vitro* growth of *B. bovis*, *B*. *bigemina*, and *B*. *caballi* and synergistic effects on that of *T. equi*, respectively, and the combination chemotherapy with low-dose regimens of CF and DA has a more potent inhibitory effect on *B. microti* in mice than do their monochemotherapies. As similar mode of CF and DA actions, which is AT-rich DNA-binding activity, suggest that these drugs in combination might contribute to work together against *Babesia* parasites. All of these results suggest that CF–DA combination chemotherapy is a better choice for the treatment of animal piroplasmosis as compared to CF and DA monochemotherapies. Further evaluation of the efficacy of combination therapy for *B. bovis* and *B. bigemina* in cattle and *B. caballi* and *T. equi* in horses will be necessary for the future application of this therapy against piroplasmosis in animals. In addition, future studies should prefer to investigate combination therapy with CF and atovaquone, azithromycin, or clindamycin, which are currently available drugs for human babesiosis.

## Materials and Methods

### Ethics statement

All animal experiments were conducted in accordance with the Regulations on Management and Operation of Animal Experiments, and all animal and DNA experiments approved by the Animal Care and Use Committee and Biological Safety Committee for Pathogen, respectively, of Obihiro University of Agriculture and Veterinary Medicine, Hokkaido, Japan (accession number of animal experiment: 28–110/28–111; DNA experiment: 201325-4/201321-4). All experiments in this study were conducted in accordance with the Fundamental Guidelines for Proper Conduct of Animal Experiment and Related Activities in Academic Research Institutions under the jurisdiction of the Ministry of Education, Culture, Sports, Science and Technology, Japan.

### Parasites and animals

*In vitro* cultures of the Texas strain of *B. bovis*, the Argentine strain of *B. bigemina*, and USDA strains of *B. caballi* and *T. equi* were maintained as previously described^25–27^.

A Munich strain of *B. microti* and eight weeks old female BALB/c mice (purchased from CLEA Japan, Inc., Tokyo, Japan) were used for the animal experimental model.

### Chemicals and reagents

All chemicals and reagents were purchased from Sigma-Aldrich (Tokyo, Japan), except DA (Novartis Animal Health). CF and DA were dissolved at 10 mM in DMSO and M-QW, respectively, as stock solutions for the *in vitro* inhibition assay. The solvents of CF and DA (DMSO and M-QW, respectively) were used in control cultures at the same concentrations as those used to prepare the highest concentration of these drugs in each *in vitro* inhibition assay. For the *in vivo* inhibition assay, 10, 15, and 20 mg/kg CF and 10, 15, and 25 mg/kg DA were dissolved in sesame oil and autoclaved physiological saline (0.9% NaCl w/v, pH 7.2), respectively, just before the treatment.

### *In vitro* inhibition assay of CF–DA combination

The *in vitro* inhibition assay was performed as previously described^18,28^. Briefly, 20 μM CF and 4 μM DA were prepared in culture media from 10 mM stock solutions for the highest concentration of each drug for the *in vitro* inhibition assay of the CF–DA combination. Subsequently, 20 µM CF and 4 µM DA were used to prepare various combinations of CF and DA as previously described^18^. For example, 4/5 of 20 µM CF (16 µM) and 1/5 of 4 µM DA (0.8 µM) were used to prepare a 4:1 CF–DA combination. Similarly, CF–DA combinations were prepared at ratios of 3:2 (12 µM CF: 1.6 µM DA), 2:3 (8 µM CF: 2.4 µM DA), and 1:4 (4 µM CF: 3.2 µM DA). The initial concentrations prepared for mono (20µM CF and 4 µM DA) and combination (4:1, 3:2, 2:3, and 1:4 CF–DA) drugs were again diluted from highest to lowest (1/2, 1/4, 1/40, and 1/400) concentrations using culture media to calculate the IC_50_ for the respective proportions of CF and DA combinations as well as that for these mono-drugs. The 0.2% DMSO and 0.04% M-QW (solvents for CF and DA, respectively) were used as controls regardless of whether these solvents affect growth of parasites. The solvent concentrations were the same as those used to prepare the highest concentration of CF or DA in culture media in each *in vitro* assay. Finally, 97.5 (*B*. *bovis* and *B*. *bigemina*) or 95 µl (*B*. *caballi* and *T*. *equi*) of culture media with CF–DA combinations and controls in triplicate was added into two 96-well culture plates for each parasite, and then 2.5 (2.5% hematocrit for bovine parasites) or 5 µl (5% hematocrit for equine parasites) of 1% parasitized RBCs (as an initial parasitemia was 1%) was added to each well as previously described^28^. All cultures were incubated at 37ºC in an atmosphere of 5% CO_2_, 5% O_2_, and 90% N_2_ for 4 days without changing the media. On the fourth day of culture, the growth inhibition-relative fluorescent values were determined by a fluorescence-based assay. The experiment was repeated 3 times.

### *In vivo* evaluation of chemotherapeutic activity of CF–DA combinations

The *in vivo* inhibition assay was performed as previously described^6^. Briefly, mice (5/group) were injected intraperitoneally with 1 × 10^7^ *B. microti*–infected RBCs. Parasitemia and hematocrit were monitored every 2 and 4 days by microscopy and a Celltac α MEK-6450 automatic hematology analyzer (Nihon Kohden Corporation, Tokyo, Japan), respectively. When parasitemia over 1% was observed in all mice, groups I, II, III, IV, and V were administered 0.2 ml of sesame oil and physiological saline as an untreated control, 0.2 ml sesame oil and 25 m/kg DA, 20 mg/kg CF and 0.2 ml physiological saline, 15 mg/kg CF and 10 mg/kg DA, and 10 mg/kg CF and 15 mg/kg DA, respectively. CF and sesame oil and DA and physiological saline were administered via oral and intraperitoneal routes, respectively, for five consecutive days. Parasitemia was calculated by counting infected RBCs among 2000 RBCs using Giemsa-stained thin blood smears prepared every two days until no parasite was detected in any mice. The experiment was repeated 3 times.

### PCR detection and infectivity of treated *B. microti*

After *in vivo* inhibition assay, all mice were anesthetized, and then blood (collected by cardiac puncture), brain, heart, spleen, kidney, and liver tissues were sampled and used for DNA extraction. PCR assay was performed using as previously described^6^.

1 × 10^8^ RBCs from the groups I–V were transfused into new 5 groups of mice (3/group) by intraperitoneal injection. Parasitemia was monitored via Giemsa-stained thin blood smears prepared every three days for 36 days.

### Two-day inhibition assay and timely morphological changes and development of CF- or DA-treated *B. bovis*

Ninety microliters of GIT culture media containing CF at 0.1, 0.5, 1, 1.5, 2, 2.5, 3, and 4 µM or DA at 0.01, 0.05, 0.1, 0.25, 0.5, 1, and 2 µM, 0.04% DMSO or 0.08% M-QW was added to a 96-well culture plate in triplicate. Ten microliters of 1% parasitized RBCs (10% hematocrit) were added to each well^27,29^. The cultures were incubated for 2 days, and the media with drugs and controls were changed on days 0 and 1 of the cultures. The IC_25_, IC_50_, and IC_75_ values were determined on day 2 of the culture by microscopy and fluorescence-based assay separately as previously described method with a minor modification^27,29^. The experiment was repeated 3 times.

Morphological changes of CF- or DA-treated *B. bovis* parasites were observed at 0.5, 1, 2, 4, 6, 8, 12, 24, 36, and 48 hours in RBC smears by microscopy. The pair-and single-cell stages of the intraerythrocytic parasite were monitored in 400 iRBCs in Giemsa-stained smears. Cultures were conducted in the prior manner with a minor modification: 900 µl of GIT culture media containing CF or DA at final concentrations of IC_25_, IC_50_, and IC_75_, 0.02% DMSO or 0.03% M-QW was added into a 24-well culture plate in triplicate. A hundred µl of 1% parasitized RBCs (10% hematocrit) were added to each well. Culture media with drugs and controls were changed at 0 and 24 hours of cultures.

### One-day inhibition assay and mRNA and DNA quantification in CF- or DA-treated *B. bovis*

IC_50_ and IC_75_ of CF and DA against *B. bovis* were determined using 8 various concentrations of each drug and controls (0.4% DMSO or 0.2% M-QW) in triplicate. Ninety microliters of GIT culture media (no serum) without drugs and controls and 10 µl of 1% parasitized RBCs (10% hematocrit) were added into 96-well culture plate on day 0 of cultures as previously described method with a minor modification^27^. On day 1, the 90 µl GIT culture media were replaced by GIT culture media with CF (at final concentrations: 0.5, 1, 2, 3, 4, 10, 20, and 40 µM), DA (at final concentrations: 0.1, 1, 2, 4, 6, 8, 10, and 20 µM) or controls. The IC_50_ and IC_75_ values were determined on day 2 of the cultures by microscopy using Giemsa-stained RBC smears. The experiment was repeated 3 times.

To obtain RNA and DNA, cultivation was performed according to the 1-day inhibition assay with 2,000 µl of cultures in a 12-well culture plate containing 1,800 µl of GIT culture media and 200 µl of iRBCs. The one thousand eight hundred microliters of GIT culture media was replaced by same volume of GIT culture media containing CF or DA at the IC_50_ and IC_75_ or controls (at final concentrations: 0.15% DMSO and 0.195% M-QW) on day 1 of the cultures. Infected RBCs were harvested on day 2 of the cultures.

RNA and DNA were extracted each from 200 µl iRBCs by a TRIzol reagent (Invitrogen, CA, USA) or QIAamp DNA Blood Mini Kit (QIAGEN, Tokyo, Japan) with RNase A digestion (Takara Bio Inc., Otsu, Japan) according to manufacturer’s instructions. RNA was purified by a QIAamp RNA Isolation Blood Mini Kit (QIAGEN, Tokyo, Japan) with recombinant DNase I digestion (Takara Bio Inc., Otsu, Japan) and then subjected to cDNA synthesis using a Random Hexamers primer (Takara Bio Inc., Otsu, Japan). The specific primers (Table S2) were designed using nuclear (1*8S rRNA*, tubulin beta chain, and Arm/cat; GenBank accession numbers: L19077, XM_001611566, and XM_001612062, respectively), mitochondrial (*cob* and *cox3*; GenBank accession number: AB499088), and apicoplast (*tufA* and *clpC*; GenBank accession number: NC_011395) gene sequences by Primer Express® Software (Life Technologies, Thermo Fisher Scientific). The qPCR assay was conducted using a Power SYBR® Green PCR Master Mix (Applied Biosystems, Warrington, UK). The fold changes of target genes relative to the *18S rRNA* were estimated in CF- or DA-treated and DMSO- or M-QW-treated *B. bovis* as previously described^30^.

### Statistical analysis and formula of ∑FIC

The standard deviation was calculated in 3 individual experiments, and a statistically significant difference (*P* < 0.05) between untreated and treated groups was analyzed using the unpaired two-tails *t*-test.

The IC_50_ values of CF or DA were determined by curve-fitting method. Antagonistic, additive, and synergistic effects were considered as a ∑FIC index: <1 indicates synergism, ≥1 and <2 indicate additive effect, ≥2 and <4 indicate slight antagonism and ≥4 indicates marked antagonism as previously described^18^.

The ∑FIC index estimation is based on the formula given below:

$$\begin{array}{c}{\rm{\Sigma }}\text{FIC}=\frac{{{\rm{IC}}}_{50}\,{\rm{of}}\,{\rm{CF}}\,{\rm{in}}\,{\rm{a}}\,{\rm{mixture}}\,{\rm{for}}\,{\rm{a}}\,\text{combination}\,}{{{\rm{IC}}}_{50}\,{\rm{of}}\,{\rm{CF}}\,{\rm{for}}\,{\rm{mono}}}\\ \quad \quad \,\quad +\frac{{{\rm{IC}}}_{50}\,{\rm{of}}\,{\rm{DA}}\,{\rm{in}}\,{\rm{the}}\,{\rm{mixture}}\,{\rm{for}}\,{\rm{that}}\,{\rm{combination}}}{{{\rm{IC}}}_{50}\,{\rm{of}}\,{\rm{DA}}\,{\rm{for}}\,{\rm{mono}}}\end{array}$$

In the qPCR, standard deviations were calculated within 3 independently cultivated cultures of untreated or treated groups, and each run was duplicated according to the previously described method and the principle of statistical analysis^30,31^.

## Electronic supplementary material

Supplementary information
