# Supplementary material for: Chemotherapeutic efficacies of a clofazimine and diminazene aceturate combination against piroplasm parasites and their AT-rich DNA-binding activity on Babesia bovis
Source: Sci Rep. 2017 Oct 24;7:13888. doi: 10.1038/s41598-017-14304-0 (PMC5654833; doi:10.1038/s41598-017-14304-0)
Supplement: Supplementary file 1 — Supplementary information [file 41598_2017_14304_MOESM1_ESM.pdf]

Chemotherapeutic efficacies of a clofazimine and diminazene aceturate combination against piroplasm parasites and their AT-rich DNA-binding activity on *Babesia bovis*

Bumduuren Tuvshintulga[1,2], Mahmoud AbouLaila[1,3], Thillaiampalam Sivakumar[1], Dickson Stuart Tayebwa[1], Sambuu Gantuya[1,4], Khandsuren Naranbaatar[4], Aki Ishiyama[5], Masato Iwatsuki[5], Kazuhiko Otoguro[5], Satoshi Ōmura[5], Mohamad Alaa Terkawi[1,6], Azirwan Guswanto[1], Mohamed Abdo Rizk[1,7], Naoaki Yokoyama[1], Ikuo Igarashi[1]*

[1]National Research Center for Protozoan Diseases, Obihiro University of Agriculture and Veterinary Medicine, Inada-cho, Obihiro, Hokkaido 080-8555, Japan. [2]Laboratory of Molecular Genetics, Institute of Veterinary Medicine, Ulaanbaatar, Mongolia. [3]Department of Parasitology, Faculty of Veterinary Medicine, University of Sadat City, Sadat City 32511, Minoufiya, Egypt. [4]Laboratory of Arachno-Entomology and Protozoology, Institute of Veterinary Medicine, Ulaanbaatar, Mongolia. [5]Research Center for Tropical Diseases, Kitasato Institute for Life Sciences, Kitasato University, Tokyo, Japan. [6]Frontier Research Center for Advanced Material and Life Science, Department of Orthopedic Surgery, School of Medicine, Hokkaido University, Kita 21, Nishi 11, Kita-ku, Sapporo, Hokkaido 001-0021, Japan. [7]Department of Internal Medicine and Infectious Diseases, Faculty of Veterinary Medicine, Mansoura University, Mansura 35516, Dakahlia, Egypt

*Corresponding author: Professor Ikuo Igarashi DVM, PhD,
National Research Center for Protozoan Diseases, Obihiro University of Agriculture and Veterinary Medicine,
Inada-cho, Obihiro, Hokkaido 080-8555, Japan
E-mail address: igarcpmi@obihiro.ac.jp; Tel.: +81-155-49-5641 (Fax: -5643)

Supplementary information

Table S1. Percentage of nucleic acid contents in genes of *B. bovis*.

Organelles	Genes	A	T	C	G	AT	CG	Total
Nucleus	*18S rRNA*	23%	27%	23%	27%	50%	50%	100%
	Tubulin beta chain	30%	21%	26%	23%	51%	49%	100%
	Arm/cat*	24%	30%	21%	25%	54%	46%	100%
Mitochondria	*cob*	26%	41%	14%	19%	68%	32%	100%
	cox3	31%	40%	13%	15%	71%	29%	100%
Apicoplast	*tufA*	39%	31%	18%	13%	69%	31%	100%
	clpC	43%	37%	10%	10%	79%	21%	100%

* Armadillo/beta-catenin-like repeat domain containing protein

Table S2. Primers used in this study for qPCR.

Genes	Sequences	Description
18S rRNA	GGACGCCTCGTTACTTTGAGA<br>AGGCGAAACCTGCTTGAAAC	Internal control
Tubulin beta chain	CCTTATCCCCTTCCCCAGGT<br>AGTGGTGCGAATCCGATCAT	Chromosomal gene
Arm/cat*	TTCGTGAACAGGCTGTTTGG<br>CGGGAGAGTCACCTGCGAT	Chromosomal gene
cob	GGTTGGGCAATGCGTTATTT<br>ATGTAGCATCATGAAAAAGAAGCAA	Mitochondrial gene encoding cytochrome b
cox3	TGATGGTTCAAATAGAGCAGAAGATT<br>GTTAAAGCTACCCAGATTAATTCAACAA	Mitochondrial gene of cytochrome c oxidase III
tufA	AAAATATGATAACTGGTGCTGTACAAATG<br>GCATAGGACCGTCTGTTAAAGAAATA	Apicoplast gene for elongation factor
clpC	GTTGTAAAGAGCATAAACAAGTCGTTTT<br>TGCCCCACTAGGACCACAAA	Apicoplast gene encoding chaperon protein

*Armadillo/beta-catenin-like repeat domain containing protein

Figure S1. Development stages of CF– or DA–treated *B. bovis*. Pair and single cell stages were counted within 400 iRBCs. (a) Pair-cell stage is considered to be a pear form of parasites, and single-cell stage is considered to be ring and degenerative forms of parasites. (b) Percentage of pair- or single-cell stages at 36 hours. (c) Percentage of pair- or single-cell stages at 48 hours. Asterisks indicate statistically significant ($P < 0.01$** or 0.05*) differences of percentages based on unpaired *t*-test analysis.

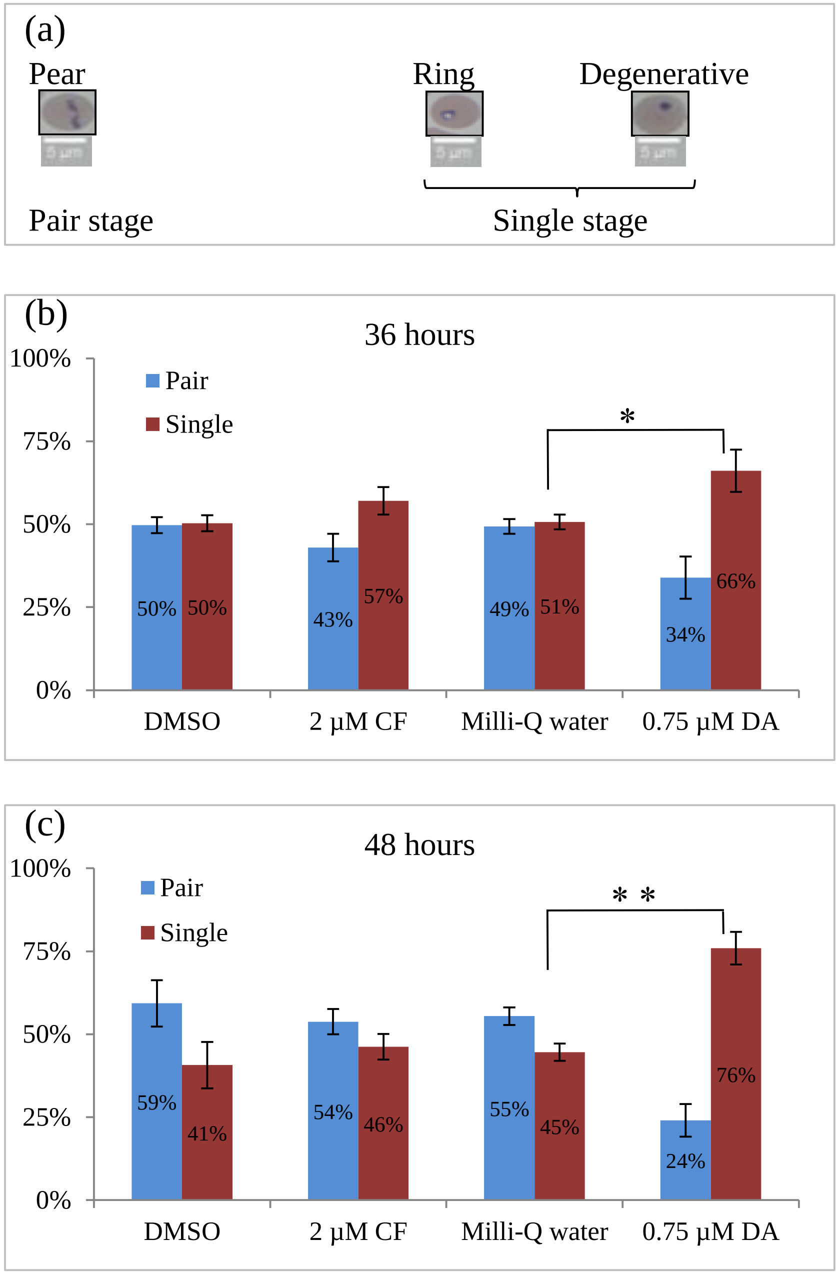

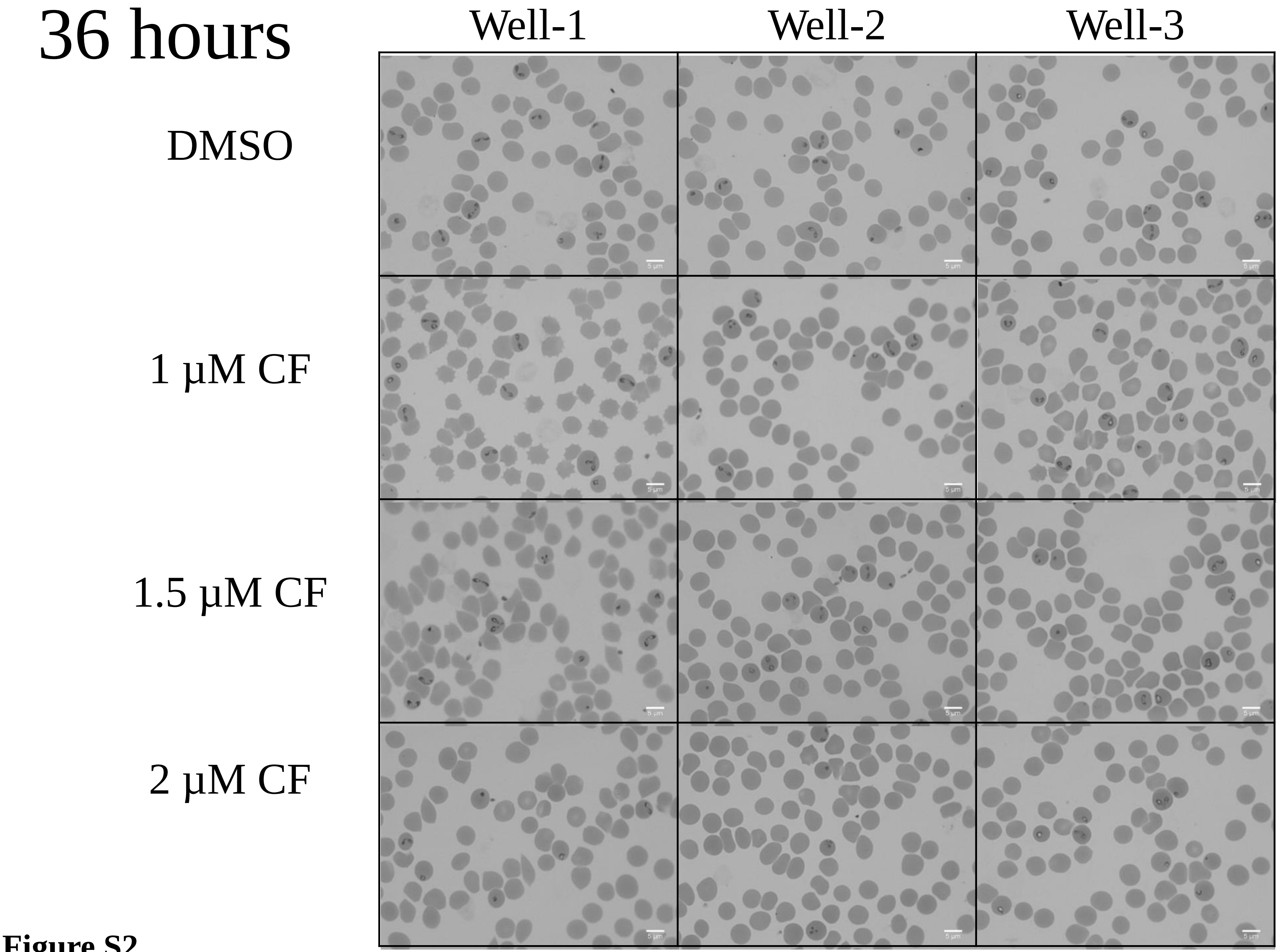

Figure S2

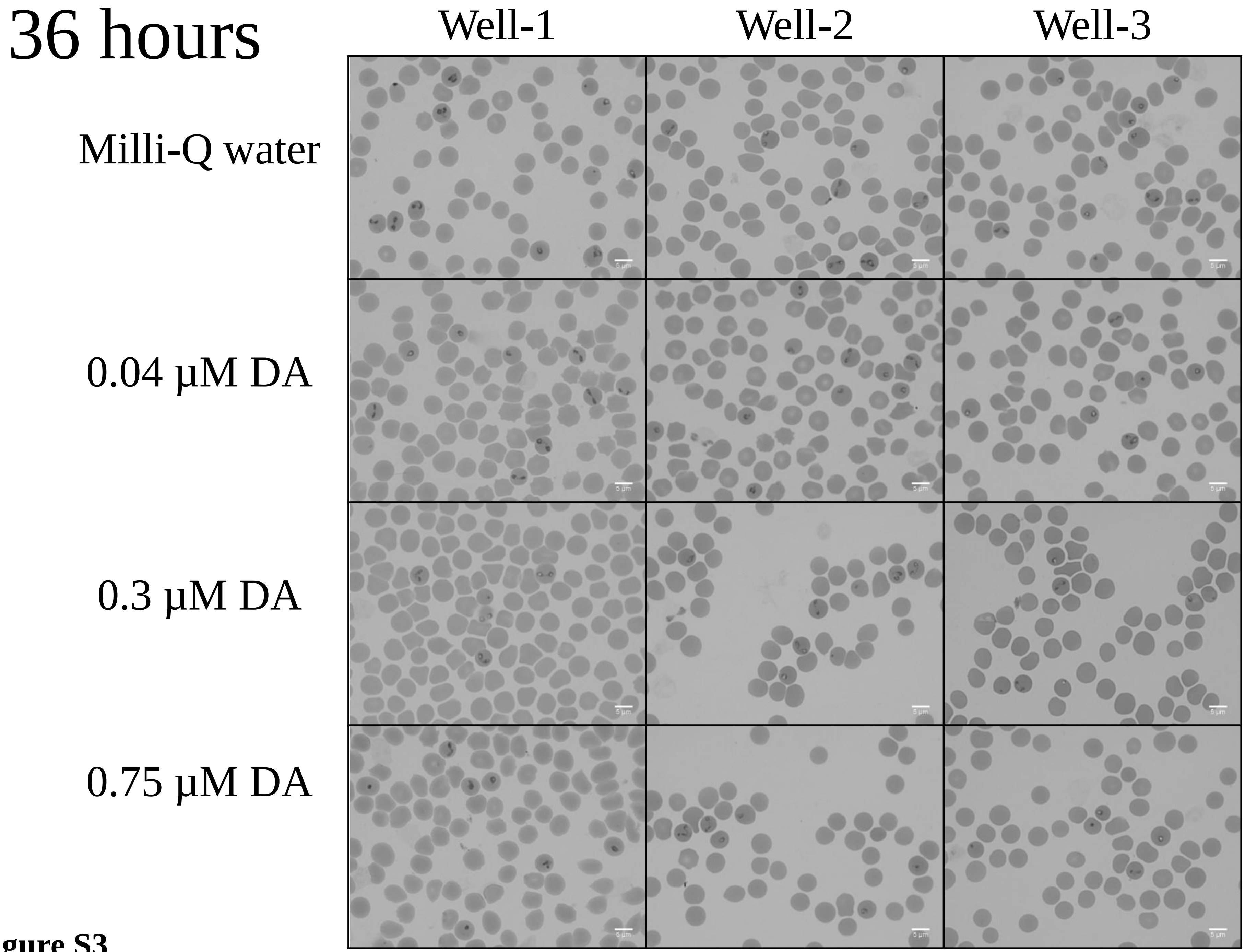
36 hours
Well-1
Well-2
Well-3
Milli-Q water
0.04 µM DA
0.3 µM DA
0.75 µM DA

Figure S3

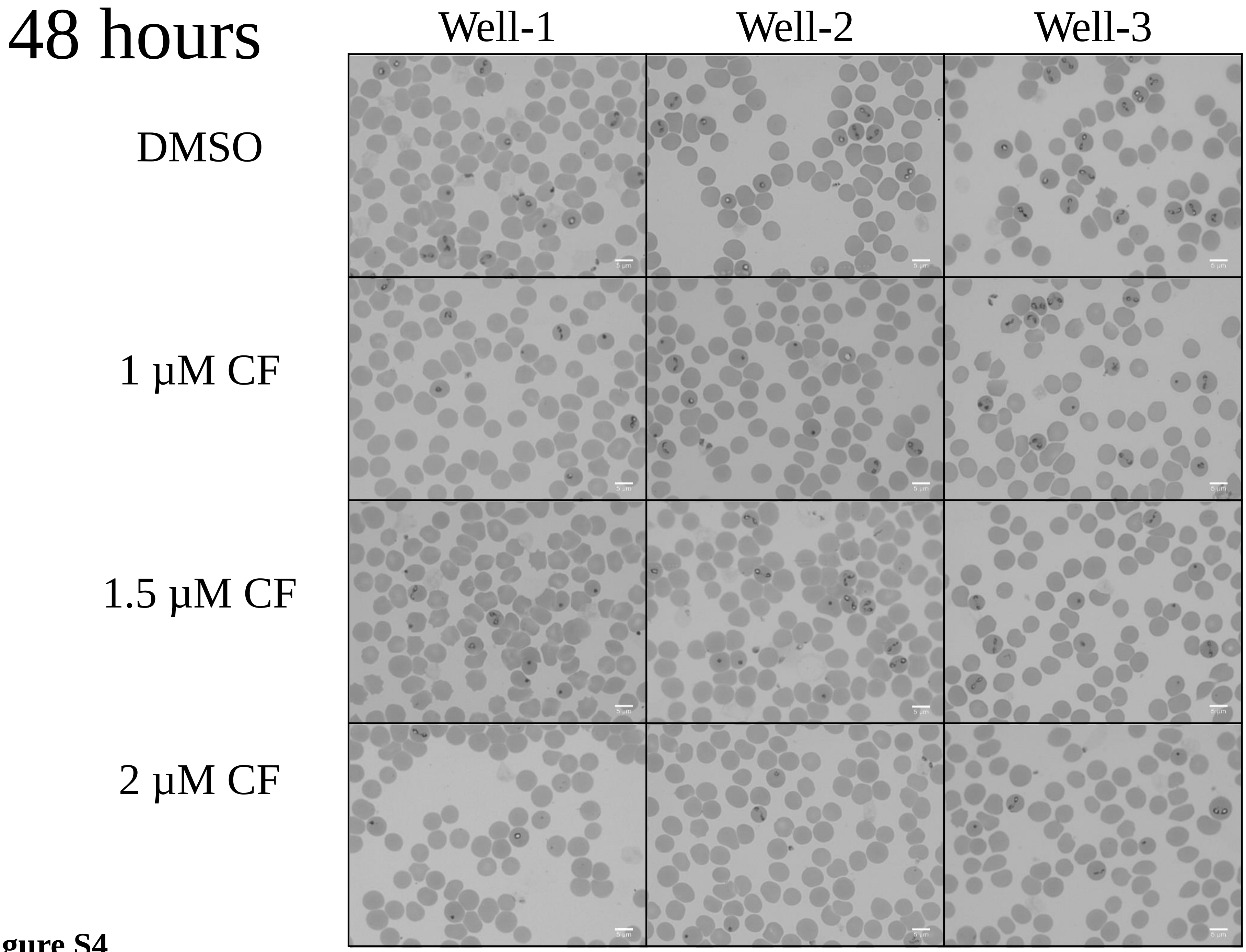

Figure S4

48 hours

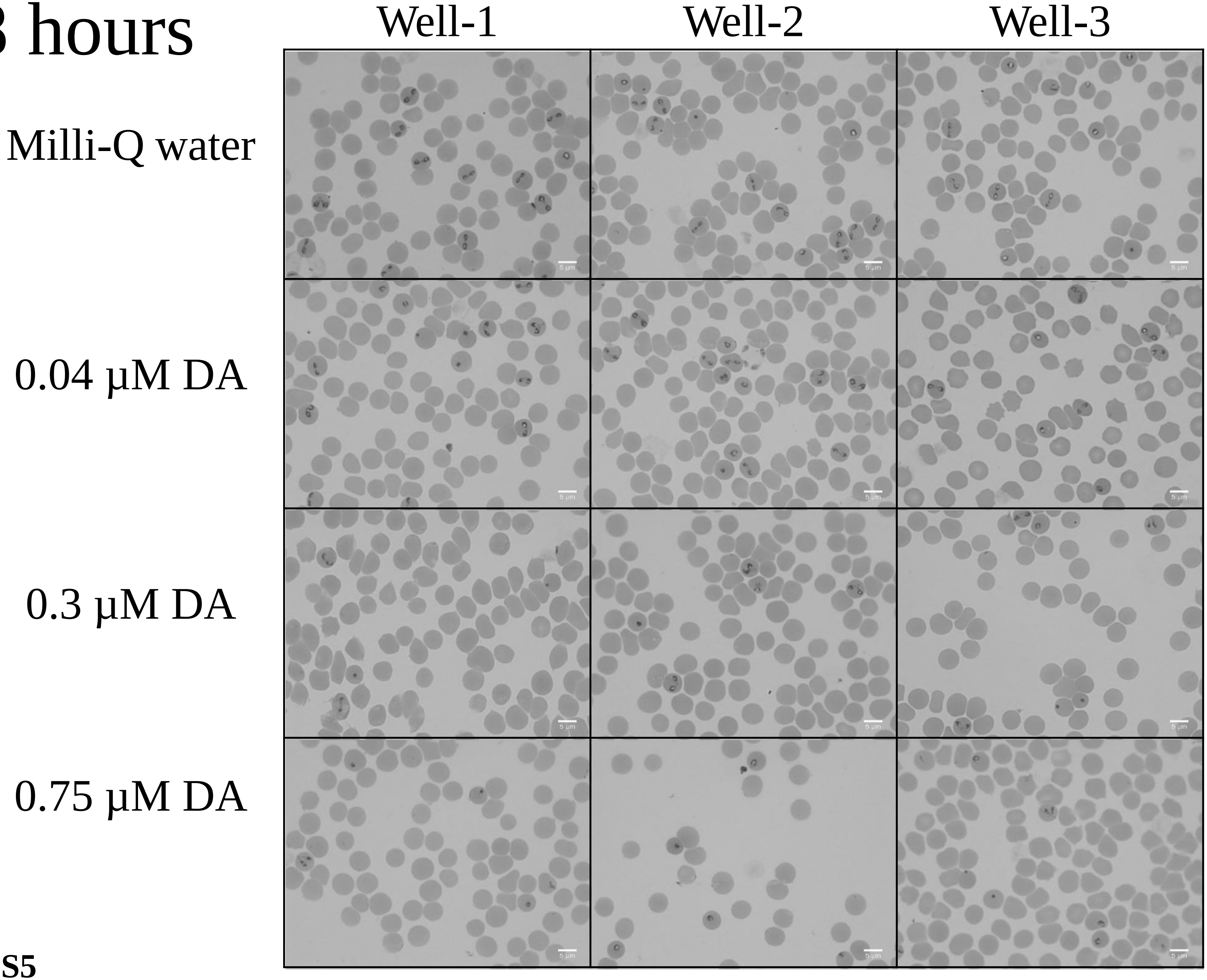

Figure S5
